# The antimicrobial efficacy of nanographene oxide and double antibiotic paste per se and in combination: part II

**DOI:** 10.1186/s12903-023-02957-5

**Published:** 2023-05-02

**Authors:** Fateme Eskandari, Yasamin Ghahramani, Abbas Abbaszadegan, Ahmad Gholami

**Affiliations:** 1 Dentist, School of Dentistry, Shiraz University of Medical Sciences, Shiraz, Iran; 2 Department of Endodontics, School of Dentistry, Shiraz University of Medical Sciences, Ghasrdasht Street, Shiraz, 71956-15878 Iran; 3 Biotechnology Research Center, Shiraz University of Medical Sciences, Shiraz, Iran; 4 Pharmaceutical Sciences Research Center, Shiraz University of Medical Sciences, Shiraz, Iran; 5 Department of Pharmaceutical Biotechnology, School of Pharmacy, Shiraz University of Medical Sciences, Shiraz, Iran

**Keywords:** Antibacterial agents, Antimicrobial, Biomedical, Dentistry, Double antibiotic paste, Endodontics, Nanographene oxide, Nanoparticles, Restorative dentistry, Yeasts

## Abstract

**Background:**

Finding strategies to overcome the rising trends of antimicrobial resistance against currently available antimicrobial agents has become increasingly relevant. Graphene oxide has recently emerged as a promising material due to its outstanding physicochemical and biological properties. This study aimed to validate previous data on the antibacterial activity of nanographene oxide (nGO), double antibiotic paste (DAP), and their combination (nGO-DAP).

**Methods:**

The antibacterial evaluation was performed against a wide range of microbial pathogens. Synthesis of nGO was achieved using a modified Hummers' method, and loading it with ciprofloxacin and metronidazole resulted in nGO-DAP. The microdilution method was utilized to assess the antimicrobial efficacy of nGO, DAP, and nGO-DAP against two gram-positive bacteria (*S. aureus* and *E. faecalis*), two gram-negative bacteria (*E. coli*, and *S. typhi*), and an opportunistic pathogenic yeast (*C. albicans*). Statistical analysis was conducted using one-sample t-test and one-way ANOVA (α = 0.05).

**Results:**

All three antimicrobial agents significantly increased the killing percent of microbial pathogens compared to the control group (*P* < 0.05). Furthermore, the synthesized nGO-DAP exhibited higher antimicrobial activity than nGO and DAP per se.

**Conclusion:**

The novel synthesized nGO-DAP can be used as an effective antimicrobial nanomaterial for use in dental, biomedical, and pharmaceutical fields against a range of microbial pathogens, including gram-negative and gram-positive bacteria, as well as yeasts.

## Background

Advancements in nanotechnology have sparked interest in the use of nanomaterials as potential antimicrobial agents [1, 2]. Their unique properties are mainly attributed to their wider surface area and more prominent quantum effects, which are inherent in nanoscale structures compared to their standard-sized counterparts [3].

Carbon-based nanomaterials encompass a variety of allotropic forms of carbon nanostructures such as graphene and carbon nanotubes [4]. Graphene is a two-dimensional single layer of carbon atoms that are covalently bonded in a hexagonal honeycomb lattice [5–7]. It serves as a fundamental building block of carbon materials of different dimensionalities including zero-dimensional buckyball and graphene quantum dot, one-dimensional carbon nanotube, and three-dimensional graphite [7, 8]. Due to its unique nanocomplex structure, graphene possesses superior physicochemical, mechanical, biological, optical, and electrical properties [9]. Graphene nanomaterials come in different formats, comprising graphene oxide (GO), reduced graphene oxide, graphene nanoribbons, graphene quantum dots, graphene nanoplates, and wrapped nanographene [10].

The most significant derivative of graphene is graphene oxide, which simulates the atomic configuration of graphene, except for the presence of epoxy and hydroxide groups on its basal plane and carboxyl groups at the edges of its skeleton [11, 12]. These functional groups augment the dispersibility of GO [9]. Notably, GO is water-soluble, thereby providing a large platform for facile functionalization-based molecule attachment [13]. Moreover, the chemistry and functionalization of the GO surface determine the hydrophilicity or hydrophobicity of the resulting structure [12].

GO possesses excellent aqueous processability, amphiphilicity, surface-enhanced Raman scattering (SERS), direct interaction with biomolecules and nanoparticles, high energy transfer efficiency, facile chemical modification, fluorescence-quenching capability, biodegradability, and biocompatibility [14–17]. These unique properties, coupled with its simple and scalable synthesis, have led to widespread application of GO in various fields including tissue engineering, biosensing, photodermal therapy, and drug delivery [15, 17].

Unlike graphene, GO is stable in various polar solutions [12]. Additionally, GO can be synthesized through a simple and cost-effective process and possesses unique physicochemical characteristics that give it promising antimicrobial properties [18, 19]. Despite this, its antibacterial mechanisms have not been thoroughly elucidated [20]. It has been proposed that the antimicrobial activity of GO can be attributed to several mechanisms, with the primary mechanism being the physical damage caused by the sharp edges of the structure (called nanoknives) to the bacterial cell membrane [21]. GO is also hypothesized to trap and generate oxidative stress, ultimately resulting in the inactivation of bacteria [1, 22].

Various investigations have explored the biosafety and biocompatibility of graphene and its derivatives. Pang et al. reported that graphene and GO exhibited good biosafety and antibacterial characteristics and can be safely used clinically in a certain concentration range for clinical purposes [23]. Furthermore, Chang et al. demonstrated safety of GO at cellular level by the favorable cell growth observed on GO film [24].

Intracanal medicaments such as calcium hydroxide (CH), double antibiotic paste (DAP), and triple antibiotic paste (TAP) are widely recommended for improved root canal disinfection [25, 26]. TAP (a combination of metronidazole, ciprofloxacin, and minocycline) has antimicrobial properties that can eliminate microorganisms from the farthest areas in the apical third, while also promoting the development of the dentin-pulp complex. However, to maintain the antimicrobial efficacy of TAP and prevent tooth discoloration caused by the minocycline, DAP (a combination of metronidazole and ciprofloxacin) is generally used as an optimized antimicrobial agent. Antibiotic pastes also seem to be effective when symptoms cannot be easily alleviated [27–29].

Verma et al. [30] reported significant direct antibacterial effects of DAP against biofilm bacteria obtained from an immature tooth with pulpal necrosis. Similarly, Sadek et al. [31] demonstrated that DAP was able to completely eliminate *Enterococcus faecalis (E. faecalis)* biofilm from radicular human dentin specimens. Moreover, Dewi et al. [32] found that TAP, modified TAP (at 5, 10 and 20 mg mL^−1^ of each drug), and DAP (at 10 and 20 mg mL^−1^ of each drug) were capable of significantly eliminating *E. faecalis*, while CH did not show significant efficacy. In regards to biocompatibility, Khoshkhounejad et al. [33] showed DAP and TAP had similar favorable effects at most of the tested concentrations and could be considered safe materials. Furthermore, McIntyre et al. [34] revealed that hydrogels containing 1 mg/mL DAP had remarkable direct antibacterial effects against single- and dual-species biofilms without jeopardizing mineralization nodule formation of dental pulp stem cells, alkaline phosphate activity, and viability. However, the use antibiotic combinations, as in TAP and DAP, can increase the risk of bacterial resistance even when used for a short duration [35].

The continuous emergence of drug-resistant pathogens necessitates the introduction of new antibacterial agents [36]. Combining two successful antibacterial agents to get complementary mechanisms of action has been shown to be effective [37, 38]. Our previous research demonstrated promising results for nGO-DAP as a root canal medicament [39]. However, since the focus of our previous antimicrobial study was on *E. faecalis*, it was deemed relevant to validate our earlier findings by observing the behavior of nGO-DAP against a panel of microorganisms including two gram-positive bacteria (*Staphylococcus aureus*, and *Enterococcus faecalis*), two gram-negative bacteria (*Escherichia coli*, and *Salmonella typhi*), and an opportunistic pathogenic yeast (*Candida albicans*). Therefore, this experiment was designed to assess the antibacterial and antifungal efficacy of nGO, DAP, and nGO-DAP against this panel of microorganisms.

## Methods

### Materials

Graphite powder was purchased from Tanfeng Graphene Technology Co., Ltd., Jiangsu, China. phosphoric acid (H_3_PO_4_), sulfuric acid (H_2_SO_4_), potassium permanganate (KMnO_4_), hydrogen peroxide (H_2_O_2_), Phosphate buffer saline (PBS, 10 × concentrate, BioPerformance Certified, suitable for cell culture, pH: 7.4), and hydrochloric acid were purchased from Sigma-Aldrich, Gillingham, UK. Ciprofloxacin and metronidazole were purchased from Alborzdaro, Gazvin, Iran. Ampicillin was purchased from Merck, Darmstadt, Germany. The Mueller–Hinton broth (MHB) medium was purchased from HiMedia (Mumbai, India) and double-distilled water was used in all experiment. All other chemicals were also purchased from Sigma-Aldrich.

### Preparation of antimicrobial agents

**Fig. 1:**
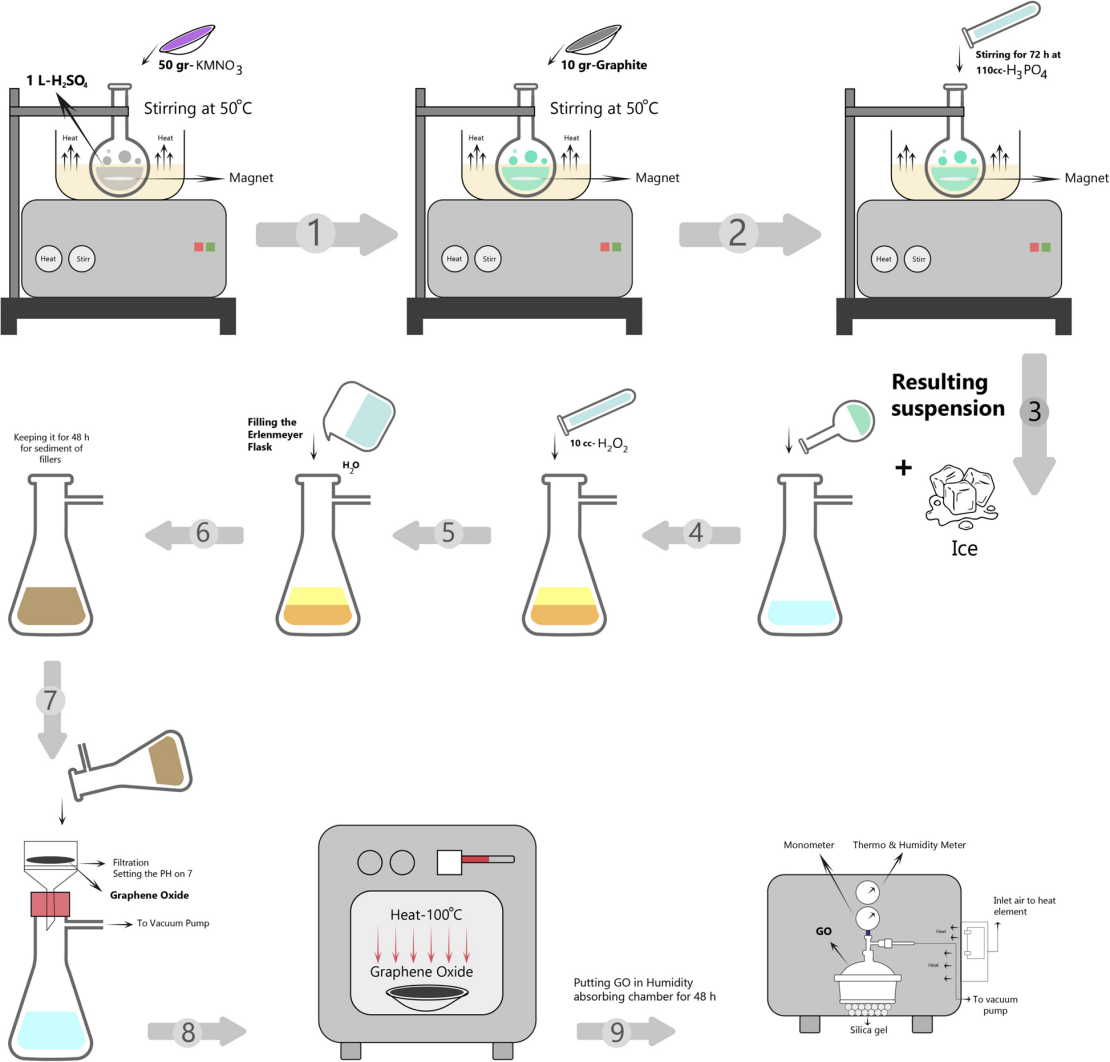
Schematic illustration of the synthesis of nGO through modified Hummer’s method. The procedure includes 9 steps: 1) combining 10 gr of Graphite flakes and 110 cc of H3PO4 and 1 L of H2SO4 (98%) 2) stirring the sample mixture for 72 h and gradually addition of KMnO4 3) removing the ice bath and stirring the sample mixture until it became pasty brownish 4) slow addition of 10 cc H2O2 5) addition of 100 mL of water 6) keeping the sample mixture for 48 h for sediment of fillers 7) filtration 8) purification 9) drying (Abbreviations: H3PO4: phosphoric acid/ H2SO4: sulfuric acid/ KMnO4: potassium permanganate/ H2O2: hydrogen peroxide)

The DAP was prepared by combining 500 mg of metronidazole and 500 mg of ciprofloxacin [40, 41]. The nGO was synthesized from graphite flakes using a modified Hummers' method as follows:

Ten grams of graphite flakes, 110 cc of H_3_PO_4_, and 1 L of H_2_SO_4_ (98%) were concreted in a 1000-ml volumetric flask that was stored in an ice bath (0 to 6 ℃) and stirred for 2 h while 50 g of KMnO_4_ was gradually added to the suspension at a cautiously controlled adding rate to keep the reaction temperature below 14 ℃. The ice bath was eliminated and the mixture was stirred at 30 ℃ until a pasty brownish mixture was obtained; stirring continued for 2 more hours while the temperature was raised every 30 min to 50 °C. Then, 100 ml of water was slowly added and the reaction temperature was rapidly raised to 96 °C by using effervescence, causing the mixture color to turn brown. The mixture was diluted with 100 ml of water while being constantly stirred. The reaction was terminated by adding 10 ml of H_2_O_2_, resulting in a yellow color. A Büchner funnel and Whatman filter paper were used and the Erlenmeyer flask was connected to a vacuum pump. The mixture was centrifuged and rinsed multiple times with 8% hydrochloride acid and then deionized water. The resulting mixture was dried in a hot air oven at 100 °C, stored in a humidity-absorbing chamber for two days, and finally GO was obtained as a powder (Fig. 1).

To load the nGO with DAP, 200 μg of ciprofloxacin, 200 μg of metronidazole, and 400 μg of an aqueous solution of nGO were mixed and incubated overnight. The resulting mixture was then centrifuged to remove any unbound DAP. The concentration of DAP in the supernatant was assessed using an ultraviolet spectrophotometer, while the drug loading was determined using Fourier-transform infrared spectroscopy (FTIR), Raman, and Atomic Force Microscopy (AFM). The loading efficiency was calculated via triplicate samples through the following formula:

$$\mathrm{Loading efficiency }= (\mathrm{A}-\mathrm{B})/\mathrm{A}$$

where A is the total concentration of DAP and B is the concentration of unbound DAP in the supernatant.

### Characterization of nanostructures

The nanostructures used in this research were characterized using Fourier transform infrared (FTIR) spectrometer (Perkin Elmer, UK), Energy-dispersive X-ray spectroscopy (EDS) (FESEM, Sigma VP, ZEISS, Germany), Transmission electron microscopy (TEM, Zeiss-EM10C-100 kV, Germany), and X-ray diffraction spectroscopy (XRD) (Siemens D5000 diffractometer, Karlsruhe, Germany).

### Antimicrobial activity assessment

*E. coli* (ATCC 11,700) and *S. typhi* (ATCC 13,311) as the gram-negative bacteria, *E. faecalis* (ATCC 25,922) and *S. aureus* (ATCC 25,923) as the gram-positive bacteria, and *C. albicans* (ATCC 10,231) fungus as the microbial microorganism were initially cultivated in Brain Heart Infusion broth overnight and then stored in 20% glycerol at -70 °C. Broth microdilution was performed to evaluate the antimicrobial activity of nGO, DAP, and nGO-DAP and determine their minimum inhibitory concentration (MIC) based on the Clinical and Laboratory Standards Institute guidelines. Serial dilutions of each antimicrobial agent were prepared in a 96-well microplate using a 90-μL Mueller Hinton Broth medium, in which, the microbial strains were cultured to achieve a turbidity of 1.5 × 10^8^ CFU/mL. Bacterial suspensions were diluted to reach 5 × 10^6^ CFU/mL, 10 μL of which was used to inoculate each microplate. Following overnight incubation at 37 °C, an ELISA reader apparatus (BioTek, Power Wave XS2) was used to measure the optical density at 600 nm. The employed positive and negative controls were ampicillin and culture media, respectively. The MIC was defined as the lowest concentration of antimicrobial agent that hindered 90% of microbial growth following overnight incubation, compared with the negative control [42].

Killing percent of antibacterial agents was calculated according to the following formula:

$$\mathrm{Killing percent}=100-[(\mathrm{ODsample}-\mathrm{ODcontrol}/\mathrm{ODbacteria}-\mathrm{ODmedia})\times 100]$$

### Statistical analysis

The data were analyzed by using SPSS software (version 25, IBM Inc., USA) through one-way ANOVA followed by Tukey’s post hoc test to compare the antibacterial agents. The significance level was set at 0.05 and the test was repeated thrice.

## Results

### Characterization of nanostructures

**Fig. 2:**
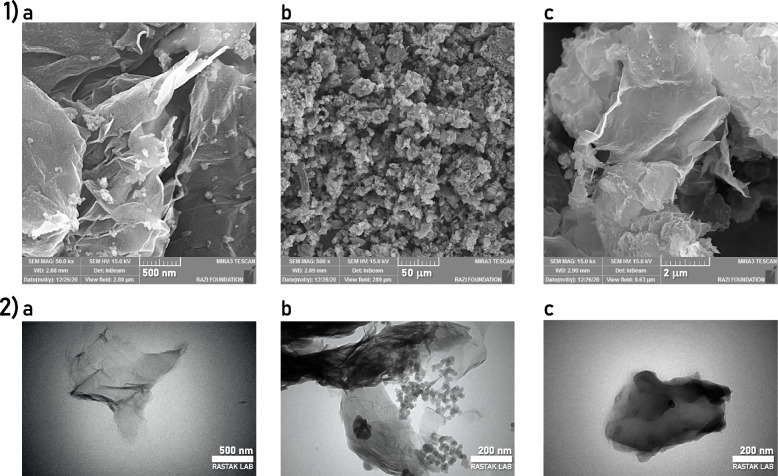
1) SEM analysis of a) nGO, b) DAP, c) nGO-DAP. The micrographs have been taken at 15.0 kV. 2) TEM images of a)nGO, b)DAP, and c)nGO-DAP

The morphology of nGO, DAP, and nGO-DAP was characterized using SEM and TEM methods (Fig. 2). The TEM graph of nGO-DAP demonstrates that DAP covered the nGO sheets and thickened the nGO (Fig. 2-c). The SEM micrographs revealed a loosely stacked and typical wrinkled structure in nGO and nGO-DAP. The wrinkled nature is essential to prevent the collapse-back phenomenon in a graphitic structure [43]. Moreover, the TEM micrographs also exhibited a wrinkled surface in nGO and nGO-DAP. As a result of exfoliation and re-exfoliation processes, this structure has been formed [44]. The outcomes of TEM analysis affirmed the contact and dispersion of DAP with and over nGO in accordance with the SEM studies.

**Fig. 3:**
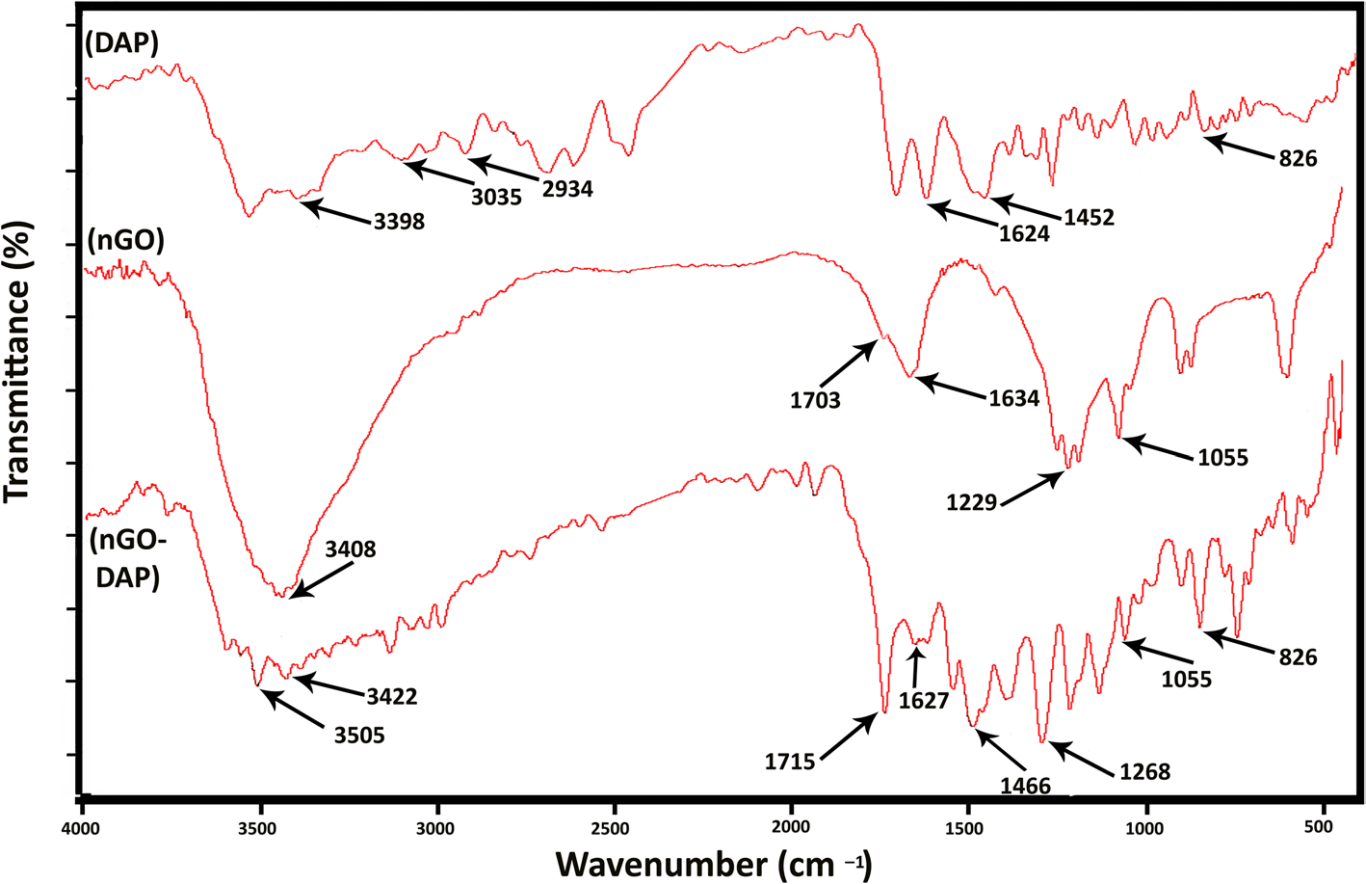
FTIR spectra of DAP, nGO, and nGO-DAP

The FTIR spectra of DAP, nGO, and nGO-DAP for wavelengths ranging 450–4000 cm^−1^ are shown at Fig. 3. The main characteristic peaks of nGO is consistent with previous studies [45–48]. The existence of carbonyl, alkoxy, and epoxy functional groups was confirmed by the FTIR peaks observed at 1703 cm^−1^, 1055 cm^−1^, and 1229 cm^−1^, respectively. The peaks at 1634 cm^−1^ and 3408 cm^−1^ are assigned to C = C benzenoid vibration and OH stretching vibration. In the FTIR spectra of DAP, the characteristic peaks observed at 826, 1452, 1624, 2924, 3035, and 3398 are attributed to ring torsion band, N = O, C = C vibration, (CH) aliphatic stretching, (CH) aromatic vibration, and NH stretching vibration, respectively, which are consistent with previous studies [49, 50]. The FTIR spectra of nGO-DAP contains the characteristic peaks of both components (nGO and DAP) which confirms the successful synthesis of nGO-DAP.

**Fig. 4:**
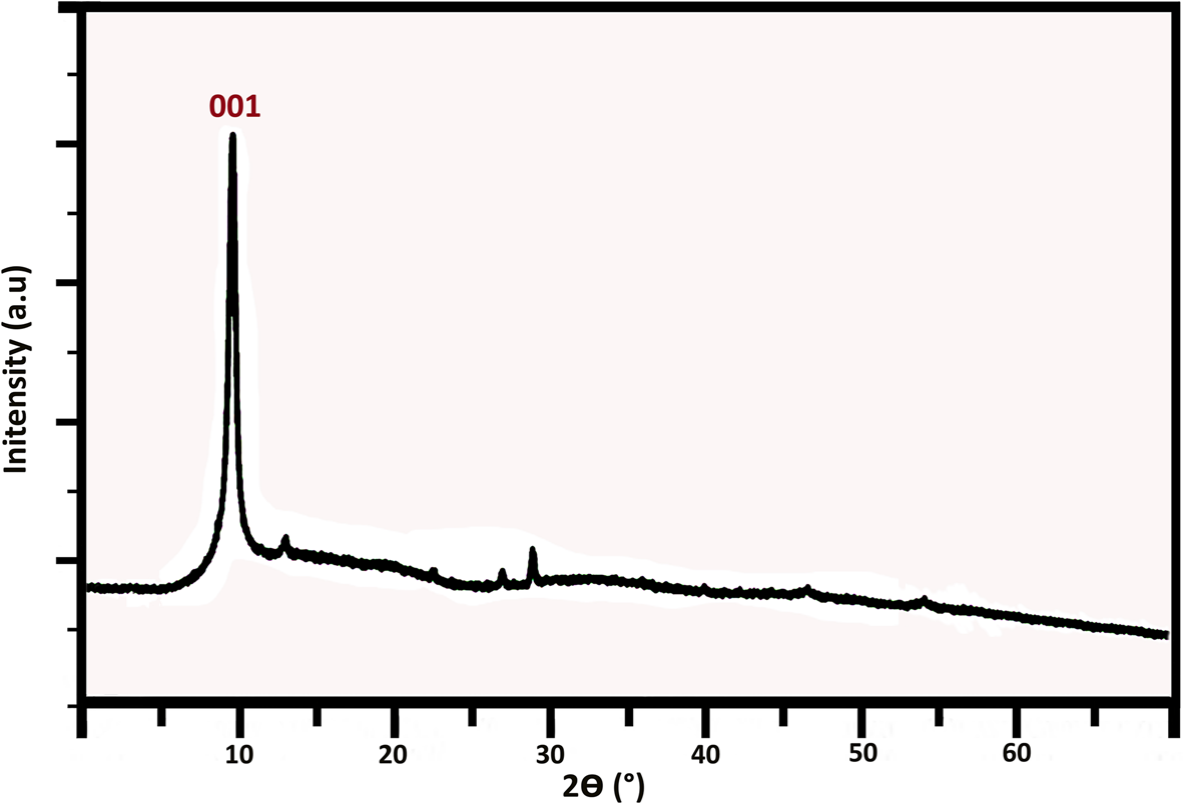
XRD pattern of nGO

Figure 4 displays the XRD pattern of nGO. The typical sharp diffraction peak of GO is observed with the diffraction peak at 2θ = 11.1°, corresponding to the (001) plane which can be attributed to the interplanar distance between nGO sheets [45, 51].

Table 1 summarizes the EDS analysis of GO, the atomic weight of which shows GO is mainly composed of 43.11 at% C, 42.24 at% O.

**Table 1:** The EDS analysis of nGO

Elt	Line	Int	Error	K	Kr	W%	A%
**C**	Ka	73.2	50.4947	0.2588	0.0923	33.38	43.11
**N**	Ka	6.7	51.3294	0.0332	0.0118	7.05	7.80
**O**	Ka	191.2	52.1640	0.3312	0.1181	43.57	42.24
**S**	Ka	364.9	28.5078	0.2810	0.1002	11.53	5.58
**Mn**	Ka	40.9	0.7011	0.0957	0.0341	4.47	1.26
				1.0000	0.3565	100.00	100.00

### Antimicrobial activity assessment

**Fig. 5:**
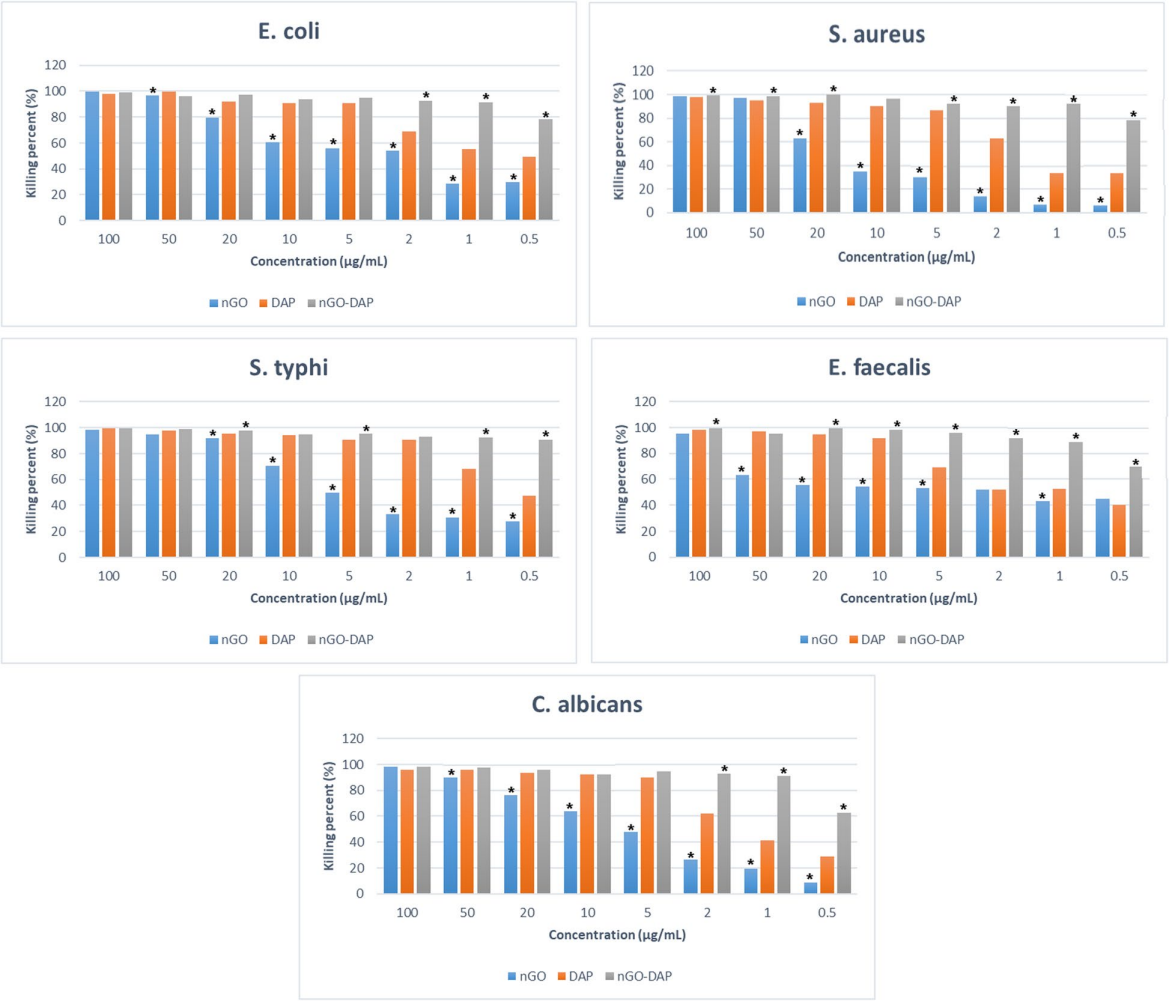
Killing percent of E. coli, S. aureus, S. typhi, E. faecalis, C. albicans after being treated with different concentrations of the experimental agents (*Statistically significant differences between DAP and other groups)

Table 2 presents the MIC values of the antimicrobial agents against the studied microbial pathogens. The killing percents (the percentages of bacteria killed by the antibacterial agents) for all the studied agents are presented in Fig. 5. The results of one-way ANOVA revealed that all antimicrobial agents significantly increased the killing percent of microbial pathogens at all concentrations compared with the control group (*P* < 0.05). Intergroup comparisons by using Tukey’s post hoc test showed that functionalization of nGO by DAP significantly elevated its antimicrobial efficacy compared to nGO and DAP per se. The antimicrobial efficacy of nGo-DAP was higher than that of nGo and DAP per se at most examined concentrations (*P* < 0.05), with the difference being greater at lower concentrations (Fig. 5).

**Table 2:** The minimum inhibitory concentration of the studied antimicrobial agents against the microbial pathogens (µg/mL)

Antimicrobial agents Microbial pathogens	nGO	DAP	nGO-DAP
*E. coli*	50	5	1
*S. aureus*	50	10	1
*S. typhi*	20	2	0.5
*E. faecalis*	100	10	2
*C. albicans*	50	5	1

*Abbreviations: DAP* double antibiotic paste, *nGO* nano graphene oxide

## Discussion

Loading ciprofloxacin and metronidazole on nGO yielded significantly higher antimicrobial efficacy compared to nGO and DAP per se. The MIC values indicated that lower concentrations of nGO-DAP were required to kill 90% of the examined microorganisms. Furthermore, higher concentrations of pure nGO were needed for better antimicrobial activity compared to DAP and nGO-DAP.

The antimicrobial activity of graphene and its derivatives is mainly attributed to their physical potential, such as their sharp-edge structure, to rupture bacterial membranes, as well as their chemical characteristics such as the generation of oxidative stresses that disrupt the proliferation process by deactivating bacterial lipids and proteins [9, 52, 53]. Other proposed antimicrobial mechanisms of graphene nanomaterials include the entrapment and isolation of bacteria from their surroundings, the destructive extraction of phospholipid molecules, and interference with protein–protein interactions [22]. Among several influential factors, the antimicrobial efficiency of graphene is mainly determined by the lateral size, particle shape, surface modifications, the culture medium condition, and the purity of GO [54, 55].

In accordance to the FTIR spectra of nGO-DAP, the bond between DAP and nGO is probably van der Waals which is a weak interaction and could facilitate the release of antibiotics at the appropriate time. So, this weak interaction would be helpful in leaching of the nanocomponent when it’s needed. Frequently, graphene and GO become modified using non-covalent physisorption encompassing coordination bonds, hydrogen bonding, electrostatic interaction, van der Waals, and π–π stacking interaction. adsorption of surfactants or small molecules and interactions with porphyrins or biomolecules, and polymer wrapping can be applied for non-covalent functionalization [56]. Rostamian et al. reported that the mechanism of antibiotics is influenced by the van der Waals intermolecular forces. In graphene oxide nanosheets, -OH groups are polarized and capable of making a network of hydrogen bonds and van der Waals forces with GO nanosheets, with water molecules and also with antibiotics [57]. Also, Ai et al. demonstrated that the van der Waals interaction was the dominant force between GO and tetracycline molecules [58].

The present findings showed that the antimicrobial activity of nGO and nGO-DAP was stronger against the gram-negative bacteria (*E. coli* and *S. typhi*) compared to the gram-positive microorganisms (*S. aureus* and *E. faecalis*). Similarly, Jawroski et al. [59] reported that silver nanoparticles (Ag), GO, and Ag-GO were more effective against *E. coli* than *S. aureus*, *S. epidermidi*s, and *C. albicans*. Likewise, Tang et al. [60] found that GO-Ag nanocomposites were bactericidal against *E. coli* and bacteriostatic against *S. aureus* through inhibition of cell division. Different antibacterial functions of nGO and its synthesized forms are probably attributed to the structure of bacterial cell walls. The thicker peptidoglycan layer in gram-positive bacteria probably makes them more vulnerable to antimicrobial agents; in other words, it provides gram-positive bacteria with superior protection against nGO and nGO-DAP structure [59, 60].

Vi et al. [55] reported that GO-Ag nanoparticles inhibited the growth of *E. coli* and *S. aureus* more effectively than GO and Ag nanoparticles per se. However, their findings indicated the higher susceptibility of *S. aureus* to GO-Ag nanoparticle structure than *E. coli*. These results are partially in contrast with the present findings. They interpreted and rationalized their finding with the presence of an outer impermeable membrane in *E. coli* that protected the peptidoglycan layer and made this bacteria more resistant to GO-Ag nanoparticles [55, 61]. Likewise, a recent study by Wang et al. [62] elucidated the superior adsorption affinity of GO to teichoic acid, a cell wall structure in gram-positive bacteria. They also reported that the adsorption affinity of GO to teichoic acid was 27 times higher than that to peptidoglycan. Hence, the expression of autolysin-encoding genes was increased and bacterial cell death was promoted through hydrolysis of peptidoglycan in the bacterial cell wall. Pulingam et al. [63] proposed that GO inhibits gram-positive and gram-negative bacteria through different mechanisms of bacterial cell entrapment and cell membrane destruction, respectively.

The MIC values of nGO against *S. aureus* in this study were similar to those reported by Khalil et al. [64]. However, their MIC values for *E. coli* and *C. albicans* were higher, which may be due to differences in the concentration or contact time of nGO with these microorganisms.

Another interesting finding of the present study was the concentration-dependent antimicrobial manner of GO-DAP and DAP against the pathogens; that is, the concentration of antimicrobial agent was directly related to the killing percent. There are other reports in the literature about the concentration-dependent manner of GO [60, 65, 66].

In the present study, increasing the concentration of nGO and nGO-DAP from 0.5 μg/mL to 100 μg/mL increased the killing percent of *E. coli* from 29.6% to 99.6%, and 78.59% to 99.3%, respectively. In line with this study, Wu et al. [67] observed the concentration-dependent effect of GO on *E. coli* survival, where increasing the GO concentration from 125 μg/mL to 250 μg/mL led to a decrease in *E. coli* survival by 11.8%. Similarly, Moraes et al. [68] noted satisfactory antimicrobial activity of GO-Ag nanocomposite against *E. coli*. The untreated *E. coli* displayed normal rod-shaped morphology and intact membranes; however, the morphological integrity of bacteria was lost when treated with 15 mg/mL of GO-Ag. Partial disruption of the cell walls and membranes of *E. coli* was observed. Additionally, leaks of intracellular contents were observed within severely damaged *E. coli* cells.

*E. faecalis* is the species most commonly isolated from failed endodontic therapy [69]. Among the microorganisms investigated in the current study, *E. faecalis* was the most resistant to nGO (MIC = 100 μg/mL) and nGO-DAP (MIC = 2 μg/mL). Notably, the most effective antibacterial material against *E. faecalis* was nGO-DAP followed by DAP and nGO, respectively, which was consistent with Eskandari et al. study [39]. Nanda et al. [70] employed Roman spectroscopy to investigate the antimicrobial mechanism of GO against *E. faecalis*, and found it to occur through the degradation of the inner and outer bacterial cell membrane of *E. faecalis* and subsequently the release of Adenine and protein from the bacteria. Regarding the effectiveness of using GO as a carrier for gene delivery when ionically bonded to cationic polyethyleneimine (PEI) polymers, Wu et al. [71] demonstrated that GO-PEI was able to effectively deliver the ASwalR plasmid into *E. faecalis* cells with high transcription of ASwalR. The use of GO-PEI-ASwalR resulted in enhanced bactericidal effects in infected canals, diminished periapical lesion, suppressed biofilm aggregation, and remarkably decreased the expression of virulent-associated gene.

The selected antimicrobial agents were also tested against *C. albicans*. Given the resistance of this opportunistic yeast to all available antifungal agents, novel alternatives are being evaluated to promote the prevention and treatment of diseases caused by this pathogen [72]. The efficacy of currently studied antimicrobial agents against *C. albicans* was slightly lower than that against gram-negative bacteria; however, nGO-DAP demonstrated significant inhibitory activity against *C. albicans* with a MIC level of 1 μg/mL. In support of this result, Shahi et al. [73] reported that GO/fluconazole enhanced antifungal activity against *C. albicans* through DNA fragmentation, with low cytotoxicity. The antifungal efficacy of GO was also confirmed through MIC evaluation. They proposed that the interaction between the hydrophilic feature of GO and the hydrophobic Candida cell wall contributed to this increased activity. Furthermore, GO per se could not efficiently prevent the adhesion of *C. albicans* to surfaces and biofilm formation. However, promising antifungal activity was observed when GO was coated with curcumin (CU) and polyethylene glycol [29].

There was little evidence of biofilm matrix formation in GO–CU and GO–CU–PEG-10% after 24 h [74]. Presumably, GO has a similar antifungal and antibacterial mechanism, which involves the sharp edges of GO and subsequent chemical oxidation, leading to splitting of the fungal cell. Additionally, the generation of reactive oxygen species has been shown to lead to the death of fungal cell [73]. Klinke et al. [75] demonstrated the potential role of *C. albicans* in causing advanced occlusal caries in rats at a high rate. Moreover, as both *C. albicans* and *E. faecalis* have been shown to play a role in root canal and periapical infections [76], the development of the novel synthesized antimicrobial agent nGO-DAP holds significant promise for use in preventive restorative dentistry and endodontics.

## Conclusion

Based on the current findings, loading ciprofloxacin and metronidazole on nGO in an attempt to synthesize a novel antimicrobial agent, yielded nGO-DAP which was effective against a broad spectrum of microorganisms including gram-positive bacteria (*S. aureus*, *E. faecalis*), gram-negative bacteria (*E. coli*, and *S. typhi*), and a common opportunistic yeast (*C. albicans*). The results also showed that the antimicrobial effectiveness of the synthesized nGO-DAP was concentration-dependent. Furthermore, this novel antimicrobial agent exhibited higher antimicrobial activity against gram-negative bacteria, compared to gram-positive bacteria and *C. albicans*. However, further *in-vivo*, *in-vitro*, and clinical investigations are necessary to comprehensively assess the antibacterial effectiveness of nGO-DAP using other antimicrobial testing methods such as minimum bacterial concentration, minimum inhibitory bacterial concentration, and antibiofilm assay, as well as to confirm its biocompatibility before it is marketed as a novel biocompatible antibacterial product. These findings are inspiring enough to represent a pivotal novel perspective for the development of more effective antimicrobial materials. They also suggest the potential future applications of nGO-DAP in pharmaceutics, biomedicine, and dentistry.

## Data Availability

The datasets used and/or analysed during the current study are available from the corresponding author on reasonable request.

## Abbreviations

*E.** faecalis*: *Enterococcus faecalis*
*S.** aureus*: *Staphylococcus aureus*
*E.** coli*: *Escherichia coli*
*S. typhi*: Salmonella typhi
*C. albicans*: Candida albicans
TAP: triple antibiotic paste
DAP: double antibiotic paste
GO: graphene oxide
CFU: colony forming unit
TEM: transmission electron microscopy
EDS: Energy-dispersive X-ray spectroscopy
FTIR: Fourier transform infrared
XRD: X-ray spectroscopy
rGO: reduced graphene oxide
PEI: polyethyleneimine
CU: curcumin
H_3_PO_4_: phosphoric acid
H_2_SO_4_: sulfuric acid
KMnO_4_: potassium permanganate
H_2_O_2_: hydrogen peroxide
